# C﻿hief digital officers: the state of the art and the road ahead

**DOI:** 10.1007/s11301-021-00227-8

**Published:** 2021-06-07

**Authors:** Lena Kessel, Lorenz Graf-Vlachy

**Affiliations:** 1 ESCP Business School, Berlin, Germany; 2 TU Dortmund University, Dortmund, Germany; 3 ESCP Business School, Paris, France

**Keywords:** Chief digital officer, Systematic literature review, Research agenda, IS Executives, M, M1, M12, M15

## Abstract

There is a lively debate on chief digital officers (CDOs). Some practitioners stress CDOs’ critical role in recrafting digital strategies and accelerating digital transformation, while others predict their disappearance. Academics have also recently begun to study this new executive position and conducted research on CDOs which is, however, scattered across different disciplines and publication outlets. We conduct a systematic literature review that consolidates these initial gains in knowledge. Specifically, we integrate findings on theoretical lenses, research designs, and key themes in studies on CDOs and we propose a framework that organizes CDO research in three broad themes: antecedents of CDO presence, the CDO role, and consequences of CDO presence. We then build on identified gaps to develop an extensive agenda for future research on CDOs. For academic researchers, we not only offer a discipline-spanning overview of knowledge on the topic at hand but also provide useful direction towards fruitful avenues for future research on CDOs. For practitioners, we offer a summary of the current scientific literature on CDOs, including relevant insights on CDO appointments, governance, and performance consequences.

**Supplementary Information:**

The online version contains supplementary material available at 10.1007/s11301-021-00227-8.

## Introduction

Digital transformation has opened the field for chief digital officers (CDOs)—an emerging top executive position that initiates and conducts digital initiatives across organizational functions (e.g., Singh and Hess 2017; Firk et al. 2019; Kunisch et al. 2020). CDOs have begun to appear in various industries, including retail, finance and insurance, as well as manufacturing and construction (Firk et al. 2019). Also, CDOs have not only had an impact on digital transformation in companies like Starbucks, McDonalds, or L’Oréal, but also in municipal governments like the City of Vancouver, or educational institutions like Harvard University (Krigsman 2015). Worldwide, organizations across sectors keep hiring CDOs. In 2020, for example, CDOs were on the recruitment agenda of the US nonprofit and healthcare organization *Mayo Clinic,* the German branch organization of the international bank *HSBC*, or the recruitment agency *Staffio HR* in India (CDO Club 2020a, b; LinkedIn 2020).

About 15 years into the existence of the CDO position (Singh and Hess 2017), the role is still hotly debated and researchers discuss CDOs’ role profile (e.g., Singh and Hess 2017; Tumbas et al. 2017), their contribution to digital transformation (e.g., Reck and Fliaster 2018), and future prospects for the CDO position in terms of its potential disappearance or the promotion of CDOs to other top executive roles (Hansen and Sia 2015; Chhachhi et al. 2016; Haffke et al. 2016; Singh and Hess 2017; Gimpel et al. 2018). Scientific studies on CDOs are published across disciplines and outlets, including conferences and journals in the fields of information systems (IS) and management. Contributions also appeared in both practitioner-oriented and scholarly journals.

However, a comprehensive in-depth review of this highly fragmented literature on CDOs does not exist, making it difficult to build on existing findings and to identify further research questions for studies that could bring clarity to the emerging role of CDOs and their contribution to digital transformation. The only relevant extant review on IS executives includes findings from five studies on CDOs and focuses on specific facets of the CDO literature to answer the question of how IS executives contribute to organizational performance (Drechsler 2020). But this review is limited in several ways. First, it focuses on CIOs rather than on CDOs. Second, including five studies on CDOs represents only a small subsample of the studies that have been published on CDOs. Third, the review looks at IS executives’ contributions to organizational performance from the theoretical perspective of an input-mediator-outcome (IMO) framework (Ilgen et al. 2005), restricting findings to specific thematic details within the CDO literature. In sum, the extant literature review on IS executives does not provide a sufficiently comprehensive overview to remedy the current disintegrated state of CDO research.

Our paper fills this gap, analyzes and synthesizes the scattered literature on CDOs to provide a discipline-spanning overview of major theoretical lenses, research designs, and themes in CDO research. In particular, we propose a framework that organizes literature on CDOs along three broad themes: antecedents of CDO presence, the CDO role, and consequences of CDO presence. Ultimately, we build on identified gaps in the literature to develop an extensive agenda for future research on CDOs.

We thus contribute by providing a structured overview of the topic at hand as well as useful directions towards fruitful research avenues. Our research agenda can guide future studies on the management of digital transformation, offering direction for researchers by compiling the most pressing open questions, e.g., on interacting factors that lead to CDO presence, on CDOs’ role and their impact in top management teams (TMTs), as well as on contingencies that might affect CDOs’ impact on digital innovation and firm performance. In addition, our research agenda provides insights on how further studies on CDOs can use and extend theoretical perspectives on, e.g., ephemeral top executives, digital change management, management fashions, or upper echelons theory.

## Background

CDOs are top managers typically tasked with initiating, conducting, and accelerating digital transformation across industries and countries (e.g., Singh and Hess 2017; Tumbas et al. 2017; Kunisch et al. 2020). Unlike other digital executives like chief information officers (CIOs) or chief data officers, CDOs do not have a clear functional dedication and rather interact cross-functionally across business units which results in very diverse possible CDO role profiles in terms of skills and responsibilities (Kunisch et al. 2020).

Large companies like, e.g., MTV Networks or the American publisher McGraw Hill started to use the job title CDO in 2005 (Singh and Hess 2017; Kunisch et al. 2020; Seeher et al. 2020). After 2010, CDO recruitment accelerated, leading to a CDO prevalence of five percent in S&P 1500 firms in 2018 (Kunisch et al. 2020). Between 2015 and 2017, the number of CDOs increased especially in the manufacturing, construction, and finance and insurance industries. Moreover, CDOs are especially present in Germany and France, whereas fewer CDOs are appointed in the US or the Netherlands (Firk et al. 2019).

Practitioners have also noted the prevalence of CDOs and engaged in a heated debate on whether CDOs will remain a fixture in the C-suite or disappear eventually. On the one hand, numerous consulting studies and media articles stress CDOs’ potential to drive digital strategy at the top management level (Gibson 2018; Trout et al. 2019; Sagonowsky 2021; Sharma et al. 2021). Consulting firm Deloitte, for example, reports on companies’ operational improvements after appointing CDOs with strong mandates (Sharma et al. 2021) and McKinsey & Company points to CDOs’ potential as coordinators of digital initiatives during the Covid-19 pandemic (Alatovic et al. 2020). Moreover, a recent Bain Capital report features successful CDOs like Adam Brotman who introduced mobile payment to Starbucks restaurants, which helped the coffee chain become a global brand leader (Bain Capital and the CDO Club 2018).

On the other hand, some practitioners wonder about a possible disappearance of CDOs. The World Economic Forum headlined that “Chief Digital Officers are doomed to fail” (Walde 2020), referring to a consulting study that reported a slowdown in CDO appointments between 2016 and 2018, and proposed that the role might disappear in the future, once other executives take ownership of digital transformation instead of appointing somebody else (Péladeau and Acker 2019). Furthermore, online media discuss that other roles, e.g., chief transformation officers, might soon replace CDOs (Overby 2019).

In parallel to this lively debate among practitioners, researchers have also increasingly devoted attention to CDOs, as they, on the one hand, may act as “facilitators of enterprise-wide change associated with digital transformation” (Tumbas et al. 2017, p. 121) and, on the other hand, might create costs, for example in the form of internal complexity within top management teams (TMTs) (Firk et al. 2019).

To further dig into the numerous unresolved questions on CDOs’ potential in driving digital transformation, the role’s current prevalence and future diffusion, as well as CDOs’ diverse roles, we contribute to current knowledge with a structured literature review that gives an overview of what is known about this relatively new top executive role.

## Method

**Fig. 1:**
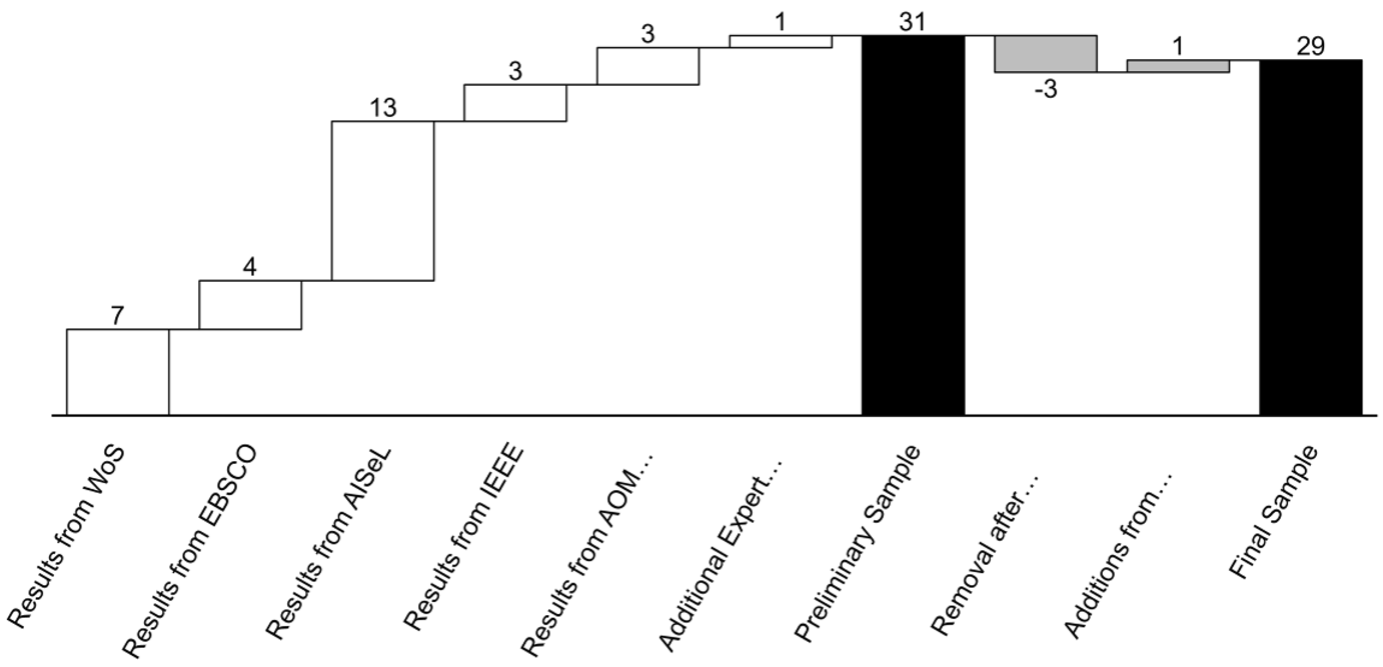
Steps of literature search and number of articles

To ensure a comprehensive account of the literature on CDOs, we followed established processes for systematic literature reviews (Webster and Watson 2002; Tranfield et al. 2003; Short 2009). To identify relevant studies, we selected “chief digital* officer*”^1^ as a keyword that had to appear in either title, abstract, or keywords of an article. We manually ensured that CDOs were in fact the focus of each article and not only, e.g., interview partners in studies on other topics. In addition, we only included English articles to make the cited literature transparent to a global English-speaking audience. We surveyed peer-reviewed papers published until July 2020. Our literature search covered a broad range of publications from IS and management journals, practitioner outlets, and conference proceedings. Following Webster and Watson (2002) and Tranfield et al. (2003), we did not limit our search to a specific set of journals because we intended to obtain a complete and up-to-date picture of the literature on CDOs. Figure 1 displays the articles we added and removed during the steps of literature research, which we further explain in the following.

First, given the multidisciplinary nature of the topic, we searched for relevant peer-reviewed journal articles in Web of Science (WoS). This allowed us to identify articles on CDOs across disciplines and yielded seven relevant references. Second, due to the high topicality of CDO research among practitioners, we also consulted EBSCO Business Source Premier (EBSCO) that lists peer-reviewed studies from practitioner-oriented journals not included in WoS. This search yielded four additional articles.

Third, considering the recent emergence of CDO research, we also included publications from major IS conferences and searched the Association for Information Systems eLibrary (AISeL)*.* Including conference proceedings allowed us to integrate the most recent findings on CDOs and extended the variety of included literature as it is suggested in relevant guidelines for systematic literature reviews (Webster and Watson 2002; Tranfield et al. 2003). After removing duplicates, irrelevant documents like interviews, theses below doctoral level, and “abstract only” papers, the search in AISeL yielded thirteen additional articles. Fourth, we consulted IEEE Xplore*,* which yielded three additional studies. Fifth, to find additional publications from the field of management, we searched the Academy of Management (AOM) Proceedings, which yielded three more studies on CDOs, for which we obtained the full texts from the authors. In addition, we added one relevant article based on an expert recommendation. Overall, we retrieved 31 articles as a preliminary sample, including research from keyword search and the recommended article.

We proceeded to read all articles and excluded two studies which did not reveal any insights on CDOs. We also excluded one literature review that did not add any new concepts or empirical evidence to the literature. Moreover, we performed a backward search and found one additional relevant article cited within our initial sample. In addition, reading through the articles revealed that authors occasionally consider executives with other titles, e.g., “digital directors” as CDOs (Firk et al. 2019). We therefore drafted a list with such titles and repeated the search with these keywords, which, however, did not yield any additional relevant studies.^2^ After this process, we arrived at a final sample of 29 articles.

During several rounds of reading, we coded each study based on central theoretical, methodological, and thematic details. Building upon this analysis, we then iteratively developed an organizing framework (Webster and Watson 2002) that synthesizes knowledge on CDOs which we discuss in the following section. Table A1 in the online appendix provides detailed data on the reviewed studies, including publication details (authors, year, database, outlet), major theoretical lenses, research designs, findings/propositions, and the central themes as identified in the organizing framework.

**Fig. 2:**
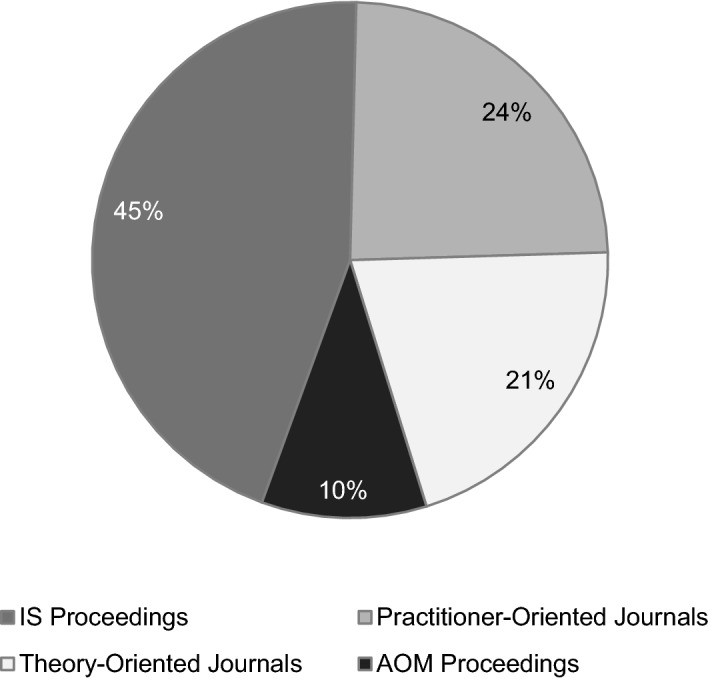
Overview of outlets

The analysis of publication details reflects the recent surge of CDO literature, with the earliest study published in 2015 (Hansen and Sia 2015). Moreover, most studies on CDOs have appeared as proceedings of IS conferences, followed by publications in practitioner-oriented journals. 21 percent of the articles in our sample appeared in theory-oriented journals and ten percent within management proceedings (AOM Proceedings) (see Fig. 2 for details).

## Organizing framework

**Fig. 3:**
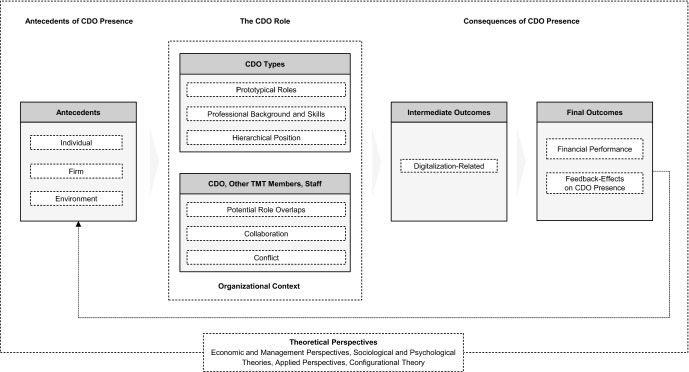
Organizing framwork for CDO research

Figure 3 displays our organizing framework, which aggregates the findings from our analysis of studies on CDOs. This framework helps not only structure extant knowledge but will also prove useful for identifying opportunities for future research. It displays the major theoretical perspectives and organizes major themes identified within the study sample.

Regarding the themes, the framework is organized in three sections. On the left, it displays the *antecedents of CDO presence,* i.e., factors that can explain how and why CDOs are appointed. More specifically, the framework differentiates between factors that relate to *individuals* (e.g., CIOs)*,* the *firm* (e.g., firm-specific organizing logics), and the *environment* (e.g., competitors)*.*

In the center, the framework displays *the CDO role*, a theme that understands different *CDO types* as actors in organizations who can assume various *prototypical roles,* possess certain *professional backgrounds and skills*, and hold varying *hierarchical positions* in the organization. Moreover, the framework includes *the CDO, other TMT members, and staff* as a sub-category of the CDO role, because CDOs’ frequent interaction with other top executives and employees shape CDOs’ role through *potential role overlaps, collaboration,* and *conflicts* that might arise. The *organizational context* that surrounds CDO types and their interaction with others illustrates contingencies, e.g., company size, that might determine CDOs’ scope of action.

The right side of the framework highlights that, not surprisingly, researchers also studied the *consequences of CDO presence,* specifically *intermediate, digitalization-related outcomes* of CDO presence, such as the initiation of new digital business processes or alliances with tech start-ups. Furthermore, scholars have investigated potential *final outcomes* of CDO presence with a focus on *financial performance.* As several scholars suggest that CDOs’ actions and performance might affect CDOs’ future presence, the framework also includes *feedback-effects on CDO presence.*

The framework also displays the various theoretical perspectives researchers used in their articles and to which we turn next.

## Theoretical perspectives in CDO research

Empirical studies look at CDOs from a variety of *economic and management* perspectives, as well as *sociological and psychological theories*. In addition, more *applied perspectives* like Havelka and Merhout's (2009) competence framework for IT professionals and *configurational theory* provide further insights into CDOs’ role and consequences of CDO presence. Table 1 presents the major coding categories for theoretical perspectives. Moreover, it includes short descriptions of the specific theoretical lenses within CDO research, including key ideas and assumptions, key representatives of the respective theories, the theory’s relevance for CDO research, the themes that are observed through the respective theory lens, and exemplary articles that view CDOs from the respective theoretical perspective.

**Table 1:** Theoretical lenses

Reference in organizing framework	Theoretical lens	Key ideas and assumptions	Key representative(s)	Relevance for CDO research	Observed Theme in CDO Research	Exemplary Articles in Study Sample
Economic and management perspectives	Contingency theory	Argues that organizational effectiveness depends on the fit of internal and external factors, e.g., strategic orientation, firm size, or environmental factors	Donaldson (2001), Child (1975)	Explains how firm-internal and external factors can lead to CDO presence and affect performance	Antecedents of CDO Presence; Consequences of CDO Presence: Financial Performance	Firk et al. (2019), Kunisch et al. (2020)
	Signaling theory	Suggests that companies can reduce knowledge asymmetries between the organization and outsiders by sending costly signals	Connelly et al. (2011), Spence (1973)	Interprets public announcements of CDO appointments as strategic signals to investors	Consequences of CDO Presence: Financial Performance	Drechsler et al. (2019)
	Upper Echelons Theory	Implies that strategic leaders’ personal characteristics, e.g., experiences, values and personality, influence organizational outcomes	Hambrick and Mason (1984), Hambrick (2007)	Proposes that CDOs’ formal education, functional experience, and general career experience, affect the amount of digital transformation activities	The CDO Role: Professional Background and Skills	Moker (2020)
	Organizational Ambidexterity	Describes the ability of organizations or individuals in organizations to balance potentially opposed objectives	Raisch et al. (2009), Gibson and Birkinshaw (2004), Turner et al. (2012)	Explores the ambidextrous role profiles of both CDOs and CIOs	The CDO Role: Potential Role Overlaps	Buchwald and Lorenz (2020), Haffke et al. (2016)
Sociological and Psychological Theories	Institutional Entrepreneurship	Proposes that actors in the role of institutional entrepreneurs break with existing institutional logics and take a central role in innovating organizations	Seo and Creed (2002), Hardy and Maguire (2008), Battilana et al. (2009), Henfridsson and Yoo (2014)	Considers the CDO as an institutional entrepreneur who enters departments with an action logic different from incumbent action logics	The CDO Role: Conflict	Tumbas et al. (2018)
	Sociological Logics of Action	Suggests that logics of actions describe symbolic constructs that influence action in a given domain, e.g., goals of organizational actors that drive their action. These logics can create routine behavior within organizations. A clash of opposite logics of action can raise conflict	DiMaggio (1997), Friedland and Alford (1991)	Explains tensions as a consequence of differing logics of action between CDO and CIO	The CDO Role: Potential Role Overlaps, Conflict	Tumbas et al. (2018)
	Theory of Transactive Memory Systems	Explains that teams succeed if they specialize in different areas of expertise, trust each other, or manage to assign tasks to respective specialists	Wegner (1987), Lewis (2003)	Explores the transactive memory system in the CDO-CIO dyad	The CDO Role: Collaboration	Horlacher (2016), Buchwald and Lorenz (2020)
	Concepts of shared understanding	Relates to individuals’ agreement on a topic	Preston and Karahanna (2009), Reich and Benabasat (2000)	Observes CDO-CIO cooperation	The CDO Role: collaboration	Horlacher (2016), Onay et al. (2018), Buchwald and Lorenz (2020)
	Role Ambiguity	Refers to a state when actors lack necessary information on their organizational position which may have a negative impact on performance	Rizzo et al. (1970), House and Rizzo (1972), Miles (1976)	Explains depressed stock prices as a consequence of mutual CDO-CIO appointments	Consequences of CDO Presence: Financial Performance	Zhan and Mu (2016)
Applied Perspectives	Role typologies for IS executives	Distinguishes between several managerial roles in the context of innovation	Grover et al. (1993), Carter et al. (2011), Mintzberg (1973), Hauschildt and Kirchmann (2001), Rost et al. (2007), Rese et al. (2013), Howell and Higgins (1990)	Distinguishes between CDOs’ multiple role profiles	The CDO Role: Prototypical Roles	Horlacher and Hess (2016), Reck and Fliaster (2018), Drechsler et al. (2018)
	Competence Framework for IT Professionals	Divides competences of IT professionals into the categories of personal, professional, business, and technical competences	Havelka and Merhout (2009)	Explores CDOs’ skillsets	The CDO Role: Professional Background and Skills	Tahvanainen and Luoma (2018)
	Vertical and Horizontal IT Governance Mechanisms	Determines the centralization of decision-making (perspective of vertical governance mechanisms) and describes structural design choices that regulate cross-functional interaction (perspective of horizontal IT governance mechanisms)	Brown (1999), Brown and Grant (2005), Brown and Magill (1994), Brown and Sambamurthy (1998)	Analyzes IT governance around CDOs	The CDO Role: Hierarchical Position, Collaboration	Horlacher et al. (2016), Singh et al. (2020), Leonhardt et al. (2018)
Configurational theory	Configurational theory	Analyzes the presence or absence of conditions that are associated with certain outcomes in organizational structures	Fiss et al. (2013), Ragin (2008)	Distinguishes between CDO types and theorize on CDO governance configurations	The CDO Role: Prototypical Roles), Consequences of CDO Presence: Intermediate Outcomes	Reck and Fliaster (2018, 2019), Leonhardt et al. (2018)

We provide further details on the links between theoretical perspectives and thematic observations in the chapter *Themes in CDO Research* below.

## Research designs in CDO research

An analysis of the studies’ research approaches revealed a large variety of mostly empirical research designs. Only a few studies employed conceptual approaches to the topic. Table 2 summarizes details on the *type of research design*, *data collection,* and *data analysis,* and indicates the *number of studies* as well as the *references* of studies that use the respective research approach.

**Table 2:** Findings on research designs

Characteristics			No. of studies	References
Type of research design	Data collection	Data analysis	29	
Qualitative	Mostly interview data	Cross-case analysis, exploratory	4	Tahvanainen and Luoma (2018), Tumbas et al. (2017), (2018), Gimpel et al. (2018)
		Cross-case analysis, descriptive	5	Becker et al. (2018), Horlacher and Hess (2016), Horlacher et al. (2016), Singh and Hess (2017), Singh et al. (2020)
		Single-case analysis, exploratory	1	Hansen and Sia (2015)
Quantitative	Mostly archival data	Regression	5	Drechsler et al. (2018), Hornuf et al. (2020), Firk et al. (2019), Kunisch et al. (2020), Moker (2020)
		Event Study	2	Drechsler et al. (2019), Zhan and Mu (2016)
	Mostly survey data	Scale development	1	Onay et al. (2018)
Mixed methods	Mostly interview data), additional survey data for validation	Cross-case analysis, exploratory	1	Haffke et al. (2016)
		Cross-case analysis, descriptive	1	Horlacher (2016)
	Survey data and interview data	No information	2	Gerth and Peppard (2016), Berman et al. (2020)
	Archival data, survey data, interview data	No information	1	Wade and Obwegeser (2019)
Qualitative comparative analysis (QCA)	Mostly survey data	Fuzzy set QCA (fsQCA)	3	Reck and Fliaster (2018, (2019), Leonhardt et al. (2018)
Delphi study	Mostly survey data	Ranking-type Delphi	1	Seeher et al. (2020)
Conceptual	Literature analysis	–	2	Buchwald and Lorenz (2020), Giebe (2019)

We identified ten qualitative studies on CDOs, of which a majority derives results mostly from interview data and cross-case analyses. One study uses a single-case analysis (Hansen and Sia 2015). Five qualitative studies employ an exploratory approach to gather first insights on CDOs (e.g., Tumbas et al. 2017; Gimpel et al. 2018). In line with Tobin (2010) who explains that descriptive case studies “seek to reveal patterns and connections, in relation to theoretical constructs, in order to advance theory development” (p. 288), we classified the five remaining qualitative studies as descriptive, because the studies examine CDOs through a theoretical lens, e.g., by building their data analysis on governance perspectives (Singh et al. 2020).

Out of eight quantitative studies, seven analyze archival data from secondary sources (e.g., databases supplied by Thomson Reuters) and apply regression models or event study methodologies (e.g., Zhan and Mu 2016; Drechsler et al. 2019; Kunisch et al. 2020). The remaining quantitative study employs survey data to develop a scale that measures digital transformation strategy alignment among C-level managers (Onay et al. 2018).

Five studies rely on a mixed-methods approach. Two of these mixed-methods studies build on cross-case analysis and add survey data to validate interviewees’ responses (Haffke et al. 2016), while two other studies mix survey data and interview data (Gerth and Peppard 2016; Berman et al. 2020). One final study combines archival data, survey data, and interview data (Wade and Obwegeser 2019). However, as the latter three studies have appeared in practitioner-oriented outlets, they do not precisely describe their data analysis.

We further identified three qualitative comparative analysis (QCA) approaches that build on survey data, and employ fuzzy set QCA (fsQCA) for configurational analyses of CDOs’ presence in organizations (Leonhardt et al. 2018; Reck and Fliaster 2018, 2019). Moreover, one study employs a Delphi methodology, an approach that intends to derive consensus from experts’ opinions on a topic (Murry and Hammons 1995; Okoli and Pawlowski 2004; Seeher et al. 2020). Due to the scarce amount of performance data on CDOs, Seeher et al. (2020) derive KPIs for CDOs from several survey rounds with experts, following a ranking-type Delphi method (Schmidt 1997). Two further studies were purely conceptual in nature (Giebe 2019; Buchwald and Lorenz 2020).

Next, we analyzed empirical studies with respect to the characteristics of their samples. Our analysis revealed several tendencies regarding *firm size*, *industry,* and *geography,* as summarized in Table 3. Bold letters indicate major sample characteristics and the sum of papers that relate to the major characteristics accordingly. Papers with no information on the respective characteristics are not included.

**Table 3:** Summary of data samples in empirical studies

Sample characteristics	No. of papers	References
**Firm size**	**20**	
Publicly listed/large firms	9	Drechsler et al. (2018), Drechsler et al. (2019), Firk et al. (2019), Haffke et al. (2016), Hansen and Sia (2015), Hornuf et al. (2020), Kunisch et al. (2020), Moker (2020), Zhan and Mu (2016)
Multiple firm sizes	11	Becker et al. (2018), Gimpel et al. (2018), Horlacher and Hess (2016), Horlacher (2016), Horlacher et al. (2016), Leonhardt et al. (2018), Reck and Fliaster (2018), Reck and Fliaster (2019), Singh and Hess (2017), Singh et al. (2020), Tahvanainen and Luoma (2018)
**Industry**	**24**	
Multiple industries	19	Becker et al. (2018), Berman et al. (2020), Drechsler et al. (2019), Firk et al. (2019), Gimpel et al. (2018), Haffke et al. (2016), Horlacher and Hess (2016), Horlacher (2016), Horlacher et al. (2016), Kunisch et al. (2020), Leonhardt et al. (2018), Moker (2020), Wade and Obwegeser (2019), Singh and Hess (2017), Singh et al. (2020), Tahvanainen and Luoma (2018), Tumbas et al. (2017), Tumbas et al. (2018), Zhan and Mu (2016)
Only manufacturing industry	3	Drechsler et al. (2018), Reck and Fliaster (2018), Reck and Fliaster (2019)
Only banking industry	1	Hornuf et al. (2020)
Only retail industry	1	Hansen and Sia (2015)
**Geographic Scope**	**19**	
Global	7	Berman et al. (2020), Drechsler et al. (2018), Firk et al. (2019), Gerth and Peppard (2016), Gimpel et al. (2018), Wade and Obwegeser (2019), Singh et al. (2020)
Europe or North America	7	Drechsler et al. (2019), Haffke et al. (2016), Hansen and Sia (2015), Hornuf et al. (2020), Reck and Fliaster (2018), Reck and Fliaster (2019), Tahvanainen and Luoma (2018)
Europe, North America, Australia, and South America	1	Tumbas et al. (2017)
Only USA	3	Kunisch et al. (2020), Leonhardt et al. (2018), Zhan and Mu (2016)
Only Germany	1	Moker (2020)

Regarding firm size, scholars either observe publicly listed or large firms, or indicate that they analyze data from firms of various sizes, ranging from, e.g., companies with 30 employees to companies with more than 5000 employees within one study sample (Reck and Fliaster 2018). We did not identify any study on only small firms. In addition, we observed that most studies analyze data samples from companies across multiple industries. A minority of five studies explicitly observes one distinct industry, i.e., manufacturing, banking, or retail. Regarding the geographic scope of the study samples, researchers frequently collect data in global, European, or North American firms. One study adds data from Australia and South America. Studies in individual countries are rare with only four studies focusing on either the US or Germany.

## Themes in CDO research

### Antecedents of CDO presence

Antecedents of CDO presence comprise factors that concern individuals, the entire organization, or its external environment. Such factors, alone or in combination, might trigger CDO appointments (Haffke et al. 2016).

Regarding theoretical perspectives, scholars examine individual-level antecedents of CDO presence through the lens of ambidexterity (e.g., Gibson and Birkinshaw 2004; Raisch et al. 2009) which describes the ability of individuals and organizations to balance opposing objectives, for example regarding supply-side and demand-side tasks (Chen et al. 2010; Haffke et al. 2016; Buchwald and Lorenz 2020). Firm- and environment-level antecedents of CDO presence are largely observed in exploratory case studies or through the theoretical lens of contingency theory which highlights the importance of fit between governance choices and the internal and external situation of the company (e.g., Child 1975; Donaldson 2001).

#### Individual-level antecedents

Companies tend to hire CDOs to complement existing IT executives who do not sufficiently meet the expectations on digital leadership (Haffke et al. 2016; Gerth and Peppard 2016; Kunisch et al. 2020). Specifically, Kunisch et al. (2020) find a positive relation between CIO and CDO presence, suggesting that CIOs may indeed be frequently missing relevant digital leadership abilities. Building on theories of organizational ambidexterity, other scholars argue that senior managers appoint CDOs as digital strategists when the often opposing objectives of IT support and strategy development are hard to combine within the role of CIOs (Haffke et al. 2016; Buchwald and Lorenz 2020).

In addition, board composition, specifically behavioral tendencies associated with age might affect CDO presence. Scholars propose, e.g., that boards with older directors are less likely to hire CDOs because they are less open to change (Kunisch et al. 2020).

#### Firm-level antecedents

Regarding the entire firm, an increasing demand for a centralized role that coordinates digital initiatives tends to lead to CDO presence (Firk et al. 2019; Kunisch et al. 2020). Specifically, rising internal complexity due to firm size or increasing product market diversification (in terms of product portfolio and geographic scope) seems to increase the need for a CDO (Haffke et al. 2016; Singh and Hess 2017; Firk et al. 2019; Kunisch et al. 2020). Moreover, siloed or particularly busy IT and marketing departments, as well as a lack of direction for a company-wide digital strategy might further accelerate CDO appointments (Tumbas et al. 2017).

Additionally, a firm’s digitization focus area seems to impact CDO presence (Haffke et al. 2016). Firms with high dependence on easily digitized assets (e.g., media) seem to be more likely to appoint a CDO than firms in industries dependent on tangibles (e.g., mining). Hence, companies appear to be more likely to appoint CDOs if their established business model is threatened by digital alternatives (Firk et al. 2019). Moreover, firms with an external focus on digitization, e.g., in marketing, appear to be more likely to appoint CDOs than firms with an internal focus, e.g., in operations, suggesting that specific task demands related to customers or distribution channels increase the demand for CDOs (Haffke et al. 2016; Kunisch et al. 2020).

Finally, companies with declining sales tend to appoint CDOs, potentially because they hope that CDOs generate new revenue opportunities by accelerating the design of digital products and services (Kunisch et al. 2020).

#### Environment-level antecedents

Companies who hire CDOs also seem to react to market pressures, e.g., digital-savvy competitors in their industries (Haffke et al. 2016; Gerth and Peppard 2016; Singh and Hess 2017; Firk et al. 2019). In particular, the number of CDO appointments in an industry rises with the number of digital-savvy competitors (Firk et al. 2019). Scholars also propose that CDOs are likely to be a quick measure to counteract market pressures if companies perceive a strong urgency to digitize (Haffke et al. 2016; Firk et al. 2019). Moreover, several scholars suggest that the adoption of CDOs might be driven by intra-industry mimicry, i.e., imitation between companies (Drechsler et al. 2019; Firk et al. 2019; Kunisch et al. 2020).

Country-specific institutional settings that influence companies’ access to information and communication technologies (ICT), e.g., regulatory frameworks or the simple availability of ICT infrastructures likely also influence CDO presence (Firk et al. 2019). For example, less availability of ICT infrastructure can force companies to transfer their digital transformation activities to external stakeholders in other countries, and might in turn make CDO presence more likely, because companies might hire CDOs to coordinate the collaboration with external stakeholders.

### The CDO role

Scholars have studied the CDO role by investigating CDO types and CDOs’ interactions with other TMT members and staff. These studies refer to a large variety of economic and management perspectives, sociological and psychological theories, applied perspectives, as well as configurational theory (Table 1).

#### CDO types

Given that CDOs are a relatively new phenomenon, researchers have identified prototypical CDO roles (e.g., Singh and Hess 2017) with different professional backgrounds and skills (e.g., Tahvanainen and Luoma 2018). Scholars have also found that CDOs’ hierarchical position seems to vary, with some CDOs being part of the overall top management team and others working at the business unit level (Singh et al. 2020). Further, CDOs’ role appears to vary with the organizational context, CDOs are not necessarily restricted to one single role profile, and their roles can develop over time (Singh and Hess 2017; Tumbas et al. 2017; Becker et al. 2018; Kunisch et al. 2020).

##### Prototypical roles

Scholars have developed several CDO role typologies (Seeher et al. 2020) that build on diverse applied perspectives of managerial roles. Specifically, researchers relate to conceptualizations of role typologies for IS executives (e.g., Grover et al. 1993; Carter et al. 2011) that, in turn, build on Mintzberg’s (1973) managerial roles, or on roles for actors in the context of innovation (e.g., Howell and Higgins 1990; Hauschildt and Kirchmann 2001; Rost et al. 2007; Rese et al. 2013). Scholars distinguish, e.g., between the CDO in the role of an entrepreneur, spokesperson, monitor, leader, or liaison (Horlacher and Hess 2016; Singh and Hess 2017), or classify the CDO as a process promoter, relationship promoter, or innovation champion (Drechsler et al. 2018; Reck and Fliaster 2018).

To provide a structured overview of CDOs’ prototypical roles, we leverage the classification into specialist and generalist CDOs introduced by Kunisch et al. (2020), as well as CDO typologies derived from configurational analyses of CDOs’ skills, networks, and behaviors.

Specialist CDOs tend to focus on functional areas and can for example work as heads of the IT or marketing department (Kunisch et al. 2020). CDOs with the role profile of “digital marketers” for example specifically focus on data analytics to enhance the customer experience (Tumbas et al. 2017; Seeher et al. 2020). Moreover, research suggests that such specialist roles might imply low role ambiguity because CDOs can focus purely on their functional duties, e.g., marketing (Wade and Obwegeser 2019).

Generalist CDOs, in contrast, often work across departments and hierarchy levels (Kunisch et al. 2020), their requirements tend to vary considerably, and they seem to experience relatively high levels of role ambiguity (Wade and Obwegeser 2019). More specifically, extant research distinguishes different generalist CDO roles into the categories of “evangelists”, “coordinators”, and “entrepreneurs” (Singh and Hess 2017).

Evangelists promote digital initiatives across departments and hierarchies (Haffke et al. 2016; Singh and Hess 2017; Seeher et al. 2020) and have also been labeled “liaison” (Horlacher and Hess 2016). Evangelists can train employees on digital topics by, e.g., organizing workshops to foster employees’ digital expertise or by inviting experts who share their knowledge on digital transformation (Haffke et al. 2016; Horlacher and Hess 2016; Singh and Hess 2017). Evangelists can also serve as “digital advocates” (Haffke et al. 2016) for the IT function by promoting the IT function’s suggestions at the top management level (Haffke et al. 2016; Singh and Hess 2017). Moreover, evangelists often use internal and external networks, e.g., with other CDOs in the corporate group, competitors, or customers to gather ideas they can introduce to their companies (Horlacher and Hess 2016; Reck and Fliaster 2019). To measure evangelists’ performance, Seeher et al. (2020) suggest measuring how CDOs manage to spread enthusiasm for digital transformation, for example by conducting surveys that capture employees’ attitude towards digital transformation.

Generalist CDOs who coordinate digital initiatives across departments are often described as “coordinators” (Haffke et al. 2016; Horlacher and Hess 2016; Singh and Hess 2017; Tumbas et al. 2017). They can establish links between business units by initiating platforms like digital councils where managers meet to realign scattered digital initiatives (Tumbas et al. 2017). Some scholars use the terms “digital harmonizer” (Tumbas et al. 2017) or “digital orchestrator” (Haffke et al. 2016; Seeher et al. 2020) to describe similar roles. Key performance indicators (KPIs) for orchestrator CDOs can consider the alignment of departments’ digital initiatives and could, for example, measure the share of digital revenue of total revenue (Seeher et al. 2020).

Generalist CDOs in the role of “entrepreneurs” span boundaries between customer trends and process development, and adapt new products or business models, often in cooperation with CIOs (Haffke et al. 2016; Horlacher and Hess 2016; Singh and Hess 2017; Tumbas et al. 2017). Entrepreneur CDOs tend to directly engage in prototyping digital innovations, e.g., by leading digital incubators (Haffke et al. 2016) or by integrating innovative solutions into existing products like augmented reality in online shopping apps (Singh and Hess 2017). Scholars also use the terms “innovator” or “accelerator” to describe such entrepreneurial CDO roles (Haffke et al. 2016; Tumbas et al. 2017; Seeher et al. 2020). KPIs for innovators might include, for instance, the number of launched digital initiatives (Seeher et al. 2020).

Moreover, scholars additionally build on configurational theory to derive CDO types from the analysis of CDOs’ skills, networks, and behavior that seem to determine CDOs’ scope of action (Reck and Fliaster 2018, 2019). Configurational theory describes a meta-theoretical approach that builds on the notion that “the whole is best understood from a systemic perspective and should be viewed as a constellation of interconnected elements” (Fiss et al. 2013, p. 1). It tries to assess causal complexity in organizational structures by analyzing the presence or absence of conditions that are associated with certain outcomes (Ragin 2008; Fiss et al. 2013).

Scholars performing configurational analyses of CDOs’ skills, networks, and behavioral patterns identified the roles “process promoter,” “relationship promoter,” “innovation champion,” and lone icebreaker” (Reck and Fliaster 2018, 2019). Process promoter CDOs possess strong negotiation and communication skills (political skills) as well as a strong internal network. Moreover, some process promoters additionally have functional expertise in business and IT. Political skills, functional expertise, and a large internal network can empower process promoters to overcome intraorganizational hurdles and communicate new ideas, technologies, and processes across departmental boundaries. Moreover, process promoters in some cases have access to a large external network (Reck and Fliaster 2018). Reck and Fliaster (2019) refer to a similar role as “networker and catalyzer” if the CDO possesses an external network, and as “insider expert” if the CDO lacks an external network.

Relationship promoters can draw on their strong internal network, their ties to other companies in the industry, and their ties to customers to access innovative solutions which allows them to engage in innovative behavior inside their firms. Interestingly, relationship promoters seem to be successful even though they lack functional expertise. This could be explained by creativity research’s finding that the lack of functional expertise might make people more observant of external innovations (Sternberg 1988; Reck and Fliaster 2018). Some scholars further describe a similar role, namely that of “innovation evangelists” (Reck and Fliaster 2019, p.4). These are actors who, like relationship promoters, have no functional expertise in IT. However, unlike relationship promoters, innovation evangelists possess strong business expertise (Reck and Fliaster 2019).

Innovation champions possess functional expertise and gather ideas from their large external networks, which can empower them to get immediately involved in the development of innovative digital products and services. For innovation champions, political skills and internal networks seem to be absent or play an insignificant role (Reck and Fliaster 2018).

Beyond these three roles, researchers additionally conceptualized “lone icebreaker” CDOs. These IT experts are directly involved in the innovation process but possess both poor interpersonal skills and poor networking ability (Reck and Fliaster 2019).

##### Professional background and skills

CDOs’ professional background and skills are mostly studied in exploratory case studies or through, e.g., the competence framework for IT professionals suggested by Havelka and Merhout (2009).

Overall, researchers find that leading digital transformation requires multidisciplinary professional skills in business and IT, as well as various soft skills (Singh and Hess 2017; Tahvanainen and Luoma 2018; Reck and Fliaster 2018, 2019; Berman et al. 2020). Moreover, needed skills seem to differ between specialist and generalist CDOs, which should be considered when executives decide on hiring internal or external candidates (Wade and Obwegeser 2019).

Professional experience in the functional domain, e.g., IT or marketing seems especially important for specialist CDOs who face low role ambiguity, and hiring experts from outside the company represents a reasonable choice to get the best people (Wade and Obwegeser 2019). Generalist CDOs appear to especially benefit from management and soft skills that enable them to thrive in cross-functional roles with high role ambiguity (Singh and Hess 2017; Tahvanainen and Luoma 2018; Wade and Obwegeser 2019; Berman et al. 2020). As generalist CDOs need to build credibility for their cross-functional change initiatives, hiring internal candidates to fill the position of cross-functional CDOs might be the better choice (Wade and Obwegeser 2019).

Regarding soft skills in general, CDOs seem to benefit from visionary and strategic thinking as well as inspirational skills to draft digital strategies and convince employees to follow their vision for digital transformation (Singh and Hess 2017; Tahvanainen and Luoma 2018; Berman et al. 2020). Moreover, communication, negotiation, and change management skills seem necessary to implement novel digital initiatives across departments (Singh and Hess 2017; Tahvanainen and Luoma 2018; Reck and Fliaster 2018, 2019). CDOs also seem to benefit from resilience (Singh and Hess 2017), which describes the ability to withstand employees’ and TMTs’ criticism of change efforts as well as the ability to acknowledge and overcome failure by learning from mistakes (Singh and Hess 2017). Moreover, scholars highlight that CDOs need ambition to promote a digital mindset across the company (Gimpel et al. 2018).

##### Hierarchical position

Building on frameworks of *vertical governance mechanisms* (Brown and Magill 1994; Brown and Grant 2005), scholars compare CDOs with central C-suite positions at the organizational apex and CDOs with decentral positions, e.g., at the business unit level (Horlacher et al. 2016; Singh et al. 2020). A central CDO position seems reasonable if the company intends to disseminate the digital vision from the top and wants to find uniform solutions for all departments. A decentral CDO position can be beneficial if business units must adapt digital services to, e.g., specific target groups. In global corporations, for example, subsidiaries might serve differing customer groups. In this case, CDOs with a decentral position at the subsidiary level can adapt the digital strategy for the entire corporation to the specific needs of the subsidiary. In addition, a decentral CDO position can prove beneficial if business units do not manage to collaborate on a holistic digital strategy, and each business unit follows their own digital strategy. Overall, decentral CDO positions seem to be quite rare (Kunisch et al. 2020).

#### The CDO, other TMT members, and staff

The CDO role is also shaped by CDOs’ interaction with other TMT members and staff. Frequently, CDOs enter departments as new players next to incumbent top executives and collaborate with employees from diverse departments (Horlacher et al. 2016; Tumbas et al. 2018; Singh et al. 2020 ). So far, research has especially observed potential role overlaps between CDOs and CIOs. Some studies have also evaluated how IT governance, reporting mechanisms, and sociological or psychological factors shape the collaboration between CDOs and other actors. Moreover, scholars propose strategies that might prevent potential conflicts between CDOs and incumbents.

##### Potential role overlaps

Role overlaps might occur when CDOs enter departments next to established positions with responsibilities in IT, e.g., CIOs, chief technology officers, chief marketing officers (CMOs), or chief strategy officers (Tumbas et al. 2018; Singh et al. 2020). So far, research has especially observed CDOs in comparison to CIOs, which has led to diverging findings on potential role overlaps between both top executives. Some scholars find that CDOs and CIOs perform similar tasks, depending on contextual factors such as professional backgrounds (Hansen and Sia 2015; Gerth and Peppard 2016; Gimpel et al. 2018). Hansen and Sia (2015), for example, propose that CIOs who have business and marketing expertise can fulfill the same roles as CDOs. Others build on sociological theories and argue that CDOs and CIOs follow distinct logics of action (Friedland and Alford 1991; DiMaggio 1997), with CDOs engaging in demand-side tasks with a focus on digital strategy development and customers, and CIOs rather focusing on supply-side tasks and the technological aspects of digital innovation (Horlacher and Hess 2016; Singh and Hess 2017; Tumbas et al. 2018). Overall, the rich discussion on CDOs’ and CIOs’ potential role overlaps further highlights diverging ideas of the CDO and the CIO role.

Observing the CDO and CIO roles through the lens of organizational ambidexterity (e.g., Birkinshaw and Gibson 2004; Turner et al. 2013) may help explain the heterogeneity in conceptualizations of CDO and CIO role profiles (Buchwald and Lorenz 2020). Studying CDOs’ and CIOs’ individual ability of performing tasks in an ambidextrous way, scholars argue that varying levels of CDOs’ and CIOs’ ambidextrous role orientations may have led to overlapping descriptions of CDO and CIO role profiles (Buchwald and Lorenz 2020).

Moreover, taking a historical perspective on the CIO and the CDO roles might further explain differences and similarities between CDOs and CIOs. Since the 1990s, CIOs have increasingly faced the challenge of handling supply- and demand-side tasks simultaneously, which might have eventually become overwhelming. At a point, the CIO role split into two separate roles—the demand-side oriented CDO and the supply-side oriented agile IT director. In companies where CIOs still remain both demand-side and supply-side oriented and manage to deal with tasks in an ambidextrous fashion, distinctions between CDOs and CIOs might be less obvious than in companies that employ both CDOs and CIOs in the role of agile IT directors (Haffke et al. 2016; Buchwald and Lorenz 2020).

##### Collaboration

Formal and informal horizontal governance mechanisms (Brown and Sambamurthy 1998; Brown 1999) can foster the cross-functional collaboration between CDOs, TMT members, and staff involved in digital projects (Horlacher et al. 2016; Singh et al. 2020). In particular, scholars refer to applied perspectives of horizontal governance (Brown and Sambamurthy 1998; Brown 1999), which describe formal and informal structural mechanisms that regulate cross-functional collaboration, e.g., between CDOs and other TMT members who collaborate on digital projects (Horlacher et al. 2016; Singh et al. 2020). Formal mechanisms, e.g., board meetings or digital steering committees, serve as platforms where CDOs and other executives can exchange information on the progress of digital transformation (Horlacher et al. 2016; Singh et al. 2020; Buchwald and Lorenz 2020). Informal mechanisms, e.g., incubators, workshops, or webinars, are usually voluntary and can serve as tools to foster employees’ collaboration on digital projects or to disseminate information on digital topics (Horlacher et al. 2016; Singh et al. 2020).

Singh et al. (2020) propose to align horizontal governance and the CDOs’ hierarchical position with the CDO’s role profile and the scope of the company’s digital transformation strategy, i.e., whether business units follow a company-wide digital strategy or envisage business-unit specific goals for digital transformation. Centralized entrepreneur CDOs in companies with a company-wide digital transformation strategy might for example benefit from digital incubators (informal horizontal mechanism) where they can connect with employees from diverse departments and trigger cross-functional design thinking for digital innovation. In contrast, evangelist CDOs with a focus on change management might rather benefit from formal horizontal mechanisms as they must convince employees of digital innovation and rely on mechanisms that are not only voluntary.

Scholars also discuss various options of reporting structures to align CDOs’ initiatives with the board, business, and IT departments. Companies were found to establish reporting from CDOs to CEOs, or from CDOs to other TMT members, e.g., CIOs, CMOs, or chief financial officers (Becker et al. 2018; Berman et al. 2020). Moreover, CDOs seem to receive reports from several TMT members, data scientists, or other employees from IT departments, e.g., system engineers (Berman et al. 2020). Moreover, direct reporting from CDO to CEO might reflect the importance of digital transformation and can be used as a strategic signal to employees (Buchwald and Lorenz 2020). Others also propose direct reporting from the CDO to the CIO to reconcile initiatives with the IT department (Berman et al. 2020).

Several scholars explicitly used theory on transactive memory systems (Wegner 1987; Lewis 2003) and concepts of shared understanding (Reich and Benabasat 2000; Preston and Karahanna 2009) to study collaboration between CDOs and CIOs (Horlacher 2016; Onay et al. 2018; Buchwald and Lorenz 2020). For example, scholars build on theory of transactive memory systems to explain that positioning on the same hierarchical level and coordination mechanisms like regular meetings between CDO, CIO, and the board seem to encourage cooperation (Horlacher 2016; Horlacher and Hess 2016; Buchwald and Lorenz 2020). In addition, researchers argue that specialization in terms of clearly defined roles can further facilitate teamwork in the CDO-CIO dyad. If CDOs are, for example, responsible for managerial tasks and CIOs for technological tasks associated with digital transformation, roles tend to be clearly defined, reducing potential friction (Horlacher 2016). Ultimately, scholars adopt concepts of shared understanding to explain that similar professional and educational backgrounds in both business and IT can foster successful collaboration (Horlacher 2016).

##### Conflicts

Scholars studied conflicts between CDOs and incumbent executives from sociological perspectives of institutional entrepreneurship (Seo and Creed 2002; Hardy and Maguire 2008; Battilana et al. 2009; Henfridsson and Yoo 2014) and sociological logics of action (Friedland and Alford 1991; DiMaggio 1997). For instance, scholars describe CDOs as institutional entrepreneurs, i.e., change agents who break with existing logics of actions, such as established work routines in organizations, to initiate and implement organizational transformation (Tumbas et al. 2018).

Tensions between CDOs and incumbent IT executives might especially occur if CDOs follow different logics of action than incumbent players like CIOs (Tumbas et al. 2018). CDOs can be viewed as institutional entrepreneurs (e.g., Battilana et al. 2009) since they enter IT functions as new players and may follow different approaches than existing IT executives. In particular, CDOs can follow a “digital logic of action” that differs from incumbent IT executives’ logic of action in the five dimensions “focus of management control,” “value orientation,” “goal achievement,” “reference industry,” and “location in the value chain.” Regarding the focus of management control, CDOs seem to focus on new initiatives, whereas IT executives focus on the operational integration of IT infrastructure. CDOs’ value orientation centers on revenue-enhancing initiatives, whereas IT executives follow a cost-saving approach. Considering executives’ goal achievements, CDOs seem to value experimentation with novel technologies, while IT executives seem to avoid risks. Tumbas et al. (2018) further compare CDOs’ work mode to routines in the IT industry, in disruptive companies, or in start-ups, whereas IT executives’ logic resembles work environments of incumbent industrial organizations. Ultimately, CDOs seem to focus on customers, while CIOs focus on operations (Tumbas et al. 2018).

If CDOs’ digital action logic clashes with IT logics of incumbent players, following the strategies of “grafting,” “bridging,” or “decoupling” might help CDOs to overcome tensions (Tumbas et al. 2018). Grafting means that CDOs can merge knowledge from several departments to get IT and business executives to work towards a common goal. In this context, CDOs can, for example, introduce teams with mixed competences in both business and IT. Bridging refers to linking activities between siloed departments. Decoupling suggests creating separate business units, e.g., incubators, to prevent conflict by externalizing experimentation with digital technologies to separate entities.

#### CDOs’ organizational context

CDOs’ activities and responsibilities tend to vary between small and medium sized enterprises (SMEs), and large-scale enterprises (LSEs) (Horlacher and Hess 2016; Becker et al. 2018). CDOs in SMEs have greater freedom in shaping their roles whereas CDOs in LSEs have more distinct responsibilities and a more limited scope of action (Horlacher and Hess 2016; Becker et al. 2018). CDOs in SMEs, for example, rather deal with digital strategy and IT, whereas CDOs in LSEs seem responsible for digital strategy while CIOs deal with technology (Horlacher and Hess 2016).

### Consequences of CDO presence

Scholars have studied various consequences of CDO presence. Specifically, they explored both intermediate, digitalization-related outcomes as well as final outcomes of CDO presence.

#### Intermediate outcomes

Substantial research looks at CDOs’ effectiveness in triggering business digitization or digital innovation performance, indicating companies’ progress in effectively using, developing, and commercializing digital processes, products, and services (Leonhardt et al. 2018; Reck and Fliaster 2018, 2019). CDOs seem to especially contribute to digital innovation if existing organizational bodies are not in charge of digital projects (Leonhardt et al. 2018). CDOs can, for example, introduce a clear digital strategy and push digital innovation by creating alliances between banks and startup firms that offer technology-driven financial services and novel service packages (so-called “fintechs”) (Hornuf et al. 2020). Moreover, CDOs may positively influence sourcing of external knowledge and internal reorganization—factors that positively mediate digital innovation performance. Additionally, CDOs’ prior experience in the organization, as well as prior industry experience, may positively moderate the effect of knowledge sourcing and internal reorganization (Drechsler et al. 2018).

Furthermore, researchers using configurational analyses (Table 1) explored the effectiveness of different CDO types (determined by their skills, networks, and behaviors) in four situations of various degrees of influence (in terms of CDOs’ decision-making authority) and differing degrees of competitive pressure in the industry (Reck and Fliaster 2018, 2019). First, process promoters can use their large internal networks and professional skills to assemble more authority within the company and increase their decision-making authority if CDOs’ influence is low. If such CDOs additionally have large external networks, they can also gather ideas from outsiders to pioneer digital initiatives, even if companies are not pressured to do so.

Second, process promoters, who tend to engage in supporting others, can also use their skills and networks in contexts of low influence and high pressure. However, process promoters who get easily distracted by external networks might not be the optimal solution in situations of high pressure, as they might lose sight of internal needs.

Third, relationship promoters or process promoters can be adequate solutions in situations of high influence and low pressure. As the main barrier to initiate digital transformation initiatives in such contexts may be a lack of TMT interest in a digital agenda, relationship and process promoters with external networks can gather outside ideas and use their political skills and high influence to put digitization on the company’s agenda.

Fourth, innovation champions may be a good bet in situations of high pressure and high influence. Their access to competitors’ advances and their expertise may help them reduce employees’ anxiety about digital transformation, a factor that often impedes the adoption of digital initiatives. Moreover, innovation champions’ large internal network can help spread knowledge on digital innovation across the company if the company has not yet managed to implement transformational processes.

#### Final outcomes

Final outcomes of CDO presence have so far been observed in terms of financial performance and feedback-effects on CDOs’ persistence in organizations.

##### Financial performance

In some cases, CDOs seem to contribute to financial performance. Specifically, scholars have observed positive stock-market reactions after CDO appointments. CDO presence tends to have a positive effect on stock-market performance if the company shows high levels of dependence on intangibles and a high degree of internal diversification (Firk et al. 2019). Moreover, announcements of CDOs with a specialist role profile that is distinct from the CIO’s role tend to function as a positive signal to investors (Drechsler et al. 2019). In addition, Berman et al. (2020) propose that adequate reporting structures and the ability to align IT with business strategy further foster CDOs’ performance effects in terms of a higher return for digital investments.

However, scholars also find that CDOs do not necessarily contribute to financial performance in all cases. Taking a contingency perspective, researchers showed that a high number of digital entrants in the industry and low external digital readiness (e.g., no adequate digital infrastructure in the country of operation) weaken the effect of CDO presence on firm performance (Firk et al. 2019). Adopting signaling theory (Spence 1973; Connelly et al. 2011), scholars propose that CDO appointments which reflect mimicry rather than a well-considered strategic choice, even seem to be perceived negatively by investors (Drechsler et al. 2019). Moreover, scholars refer to theory of role ambiguity (Rizzo et al. 1970; House and Rizzo 1972; Miles 1976) to suggest that role overlaps between CDOs and CIOs can negatively affect share prices (Zhan and Mu 2016).

##### Feedback effects on CDO presence

Several scholars propose feedback effects of CDOs’ actions and performance on CDOs’ future role. Some CDOs might eventually disappear or get promoted to further business executive roles (Hansen and Sia 2015; Haffke et al. 2016; Singh and Hess 2017; Gimpel et al. 2018). Especially CDOs who primarily educate others on digital topics are suggested to potentially become obsolete once the majority of executives possesses digital skills (Haffke et al. 2016). Moreover, the organizational context, such as geography, industry, business model, or the existence of other digital leaders, might determine whether CDOs remain in organizations (Tumbas et al. 2017; Gimpel et al. 2018; Giebe 2019). Giebe (2019), for example, proposes that CDOs are at best a part of the solution for digital transformation in German banks, because CDOs alone are not likely to digitally transform entire organizations. Gimpel et al. (2018) suggest that other “CxOs” can equally perform the role of digital strategists. Ultimately, Tumbas et al. (2017) state that whilst “executive leadership for digital innovation will be needed in one form or another” (Tumbas et al. 2017, p.133), the CDO role might merge into related roles like the ones of chief innovation officers.

## Agenda for future research

This paper synthesizes current knowledge on CDOs to derive avenues for further research that can shed light on CDOs’ emerging and controversially discussed role. Our organizing framework proposes links between extant findings on antecedents of CDO presence, the CDO role, and consequences of CDOs’ presence in the context of digital transformation. Moreover, our review suggests that CDOs can contribute to business digitization and firm performance if their role fits organization-specific contingencies. To further our understanding of how CDOs can lead organizations through digital transformation, we encourage future researchers to extend the present body of research on CDOs.

In the following section, we thus explore opportunities to conceptually and methodologically extend research in each of the major themes identified in the organizing framework (Fig. 3). Table 4 summarizes *research opportunities* and further *research questions* that provide directions for future studies.

**Table 4:** Research opportunities and selected research questions

Research opportunity	Selected research questions
Opportunity 1: Antecedents of CDO presence	Which interacting factors lead to CDO presence? How do changes over time affect trends of CDO appointments? How will institutional developments affect CDOs’ future presence and diffusion?
Opportunity 2: The CDO role	How are education/skillsets/personality traits linked to the CDO role and CDOs’ effectiveness? Why do companies hire CDOs without IT skills and how do they contribute to digital innovation? How do CDOs contribute to digital change management? Which personality traits (e.g., empathy) do CDOs need to cope with the challenges of their cross-functional position in general and in special situations like crises? How do CDOs manage role transitions and the associated psychological challenges stemming from CDOs’ multiple roles? How can KPIs be further refined and developed?
Opportunity 3: The CDO and other top executives	How do CDOs affect functional, structural, or social dynamics in the TMT and at its various interfaces? How and when do CIO-CDO role transitions occur and what are their consequences?
Opportunity 4: Consequences of CDO presence	Are identified links between CDOs’ presence and consequences valid and causal? How do CDOs affect other organization-level outcomes, e.g., alliances, mergers and acquisitions, or changes to an organization’s structure? How do CDOs’ achievements affect their role?
Opportunity 5: Contingency factors	How do environmental contingencies like industry and geography affect CDO prevalence and effectiveness? How does organizational context affect CDOs’ role and effectiveness? How does board composition affect CDOs’ effectiveness?

### Antecedents of CDO presence

Future research can dig deeper into the complex interactions between antecedents of CDO presence on the individual, firm, and environment levels (Haffke et al. 2016; Firk et al. 2019; Kunisch et al. 2020). Further studies can, for example, address causal complexity due to multiple factors interacting, and potential bias (e.g., confirmation bias (Nickerson 1998) or endogeneity (Antonakis et al. 2010) in identified links between antecedents on several levels and CDO presence. Moreover, quantitative studies can help assess the generalizability of ideas derived from qualitative research, e.g., if CDO appointments after CIOs’ failures only appear in sporadic cases or can be considered a more general phenomenon.

In addition, studying changes in antecedents of CDO presence over time might reveal how broader trends affect CDO appointments. Fashion perspectives (Abrahamson 1991) could provide a suitable lens to explain how mimicry behavior may lead to CDO presence (Drechsler et al. 2019; Firk et al. 2019; Kunisch et al. 2020). Moreover, performing a Delphi-study on future trends regarding the development of the CDO role can be a starting-point to better understanding how experts see the future significance of CDOs and might further illuminate whether CDO tasks are likely to be absorbed by other executives, e.g., chief transformation officers. Integrating role perspectives can further provide a theoretical framework to focus on antecedents for certain CDO roles and might shed light on why some CDO types might become entrenched in organizations whilst others might disappear.

Ultimately, institutional developments during the Covid-19 pandemic may have changed expectations regarding CDOs’ future presence and diffusion. The increased implementation of digital tools for remote work (e.g., Alatovic et al. 2020; Marr 2020) has likely raised employees’ digital awareness, which could in turn diminish the need for certain CDO types that focus on “evangelizing” digital tools. Following Menz's (2012) call for more research on the rise and fall of functional TMT members during and after institutional developments, longitudinal data collections that observe CDO trends while companies are dealing with Covid-19 represent an opportunity to explore how shifts in digital awareness affect the diffusion of CDO positions.

### The CDO role

The CDO role warrants further exploration to improve our understanding of what CDOs are expected to do, what different kinds of CDOs actually do, and how effective they are. Specifically, it might prove valuable to study antecedents and consequences of specific educational backgrounds, skillsets, and personality traits in CDOs. For instance, as research has observed the creative potential of CDOs who contribute to digital innovation without an IT background (Reck and Fliaster 2018), qualitative investigations on CDOs with non-IT backgrounds might derive insights on why organizations hire CDOs without IT expertise and how such CDOs cope with challenges of digital transformation.

Further illuminating CDOs’ change management skills (Singh and Hess 2017; Tahvanainen and Luoma 2018; Reck and Fliaster 2019) offers the opportunity to learn more about CDOs’ potential to effect change related to digital transformation. Longitudinal studies on CDOs’ role and actions in the process of change management can further shed light on questions of how CDOs transform organizations and convince others of their digital agenda. Going beyond exploratory approaches, further studies can build on change management models that provide theoretical frameworks for several episodes and facets of change management (e.g., Kotter 1995; Hiatt 2006) CDOs might encounter. Moreover, a relational perspective of social ties in organizational change management (Battilana and Casciaro 2013) might reveal if and how CDOs’ cross-functional role and frequent interactions with other stakeholders provide a beneficial position for organizational change management in the context of digital transformation.

In addition, research on CDOs’ personality traits may shed light on factors that shape CDOs’ success and should be considered if firms intend to enhance the CDO role profile for specific contexts, e.g., crisis management during the Covid-19 pandemic. Similar to Gerstner et al. (2013) who propose that personality traits like narcissism affect leaders’ strategic choices in adopting technological discontinuities, further research on CDOs’ personalities might reveal how, e.g., CDOs’ empathy, resilience, or ambition (Singh and Hess 2017; Doonan 2018; Giebe 2019) contributes to decision-making in contexts of digital transformation.

Specifically, analyzing CDOs’ empathy might afford us valuable insights on how CDOs can contribute to organizational crisis management. Practitioners propose that Covid-19 calls for empathic CDOs who adjust remote work solutions to employees’ interests while continuously aligning digital initiatives with other TMT members to adapt digital processes to several stages of the Covid-19 crisis (Alatovic et al. 2020). Scholars might build on theoretical concepts that propose positive and negative effects of leaders’ empathy on organizational crisis management before, during, and after the crisis (e.g., König et al. 2018), to observe how empathic CDOs contribute or inhibit problem resolution at different stages of a crisis. Additionally, studies on how CDOs’ personality traits affect digital innovation performance can extend upper echelons perspectives (Hambrick and Mason 1984; Hambrick 2007) in CDO research.

Further, applying a micro-role transition perspective (Ashforth et al. 2000) represents an opportunity to learn about psychological consequences of CDOs’ frequent role-transitions between, e.g., entrepreneur and evangelist roles (Haffke et al. 2016; Singh and Hess 2017). Knowing about resulting effects like confusion or anxiety, may inform about personality traits that CDOs need in order to cope with such consequences and might help companies identify suitable candidates for CDO positions. Moreover, information on consequences of CDOs’ role switches might reveal whether companies should continue putting CDOs in several role profiles or should define clear CDO roles to reduce, e.g., CDOs’ anxiety.

To consider potential changes to the CDO role, further studies may also seek to empirically validate and extend the suggested KPIs for this position (Seeher et al. 2020). So far, scholars proposed differing KPIs only for evangelists, coordinators, marketers, and entrepreneurs. In addition, some of these KPIs, e.g., employees’ attitude towards a digital culture, are not yet readily operationalized and thus provide opportunities for research.

### The CDO and other top executives

Regarding the recent emergence of the CDO’s position among other top managers (e.g., Tumbas et al. 2018), future research can analyze how CDOs change established functional, structural, or social processes in TMTs because these might directly or indirectly affect business digitization and firm performance. Research on the CEO-TMT interface, defined as “the linkage and interaction between the CEO and other top managers” (Georgakakis et al. 2019, p. 1), as well as other intra-TMT interfaces is thus called for. For example, investigations into emerging agency relationships between CEOs, CDOs, and CIOs might be fruitful. CDOs seem to be hired because CEOs expect them to be more effective in digital transformation than CIOs (Haffke et al. 2016). Agency theory (Eisenhardt 1989) might provide a suitable lens to study relations and problems between CEOs and CDOs, to compare differences between CEO-CDO and CEO-CIO relations, and to observe performance effects of emerging agency relationships related to CDO presence. In addition, viewing the CDO-CIO dyad from a pooled leadership perspective—a theoretical lens that sheds light on dynamics of joint leadership at top management level in organizations across industry sectors (Denis et al. 2012) —can reveal more about the dynamics of plural leadership in IS contexts. Specifically, a pooled leadership lens may inform about contingencies that favor CDO-CIO dyads instead of solo leadership structures and might reveal disadvantages and opportunities of CDO-CIO constellations.

In addition, observing CIO-CDO role transitions (Haffke et al. 2016) from a segmented role transition perspective (Ashforth et al. 2000) could provide an opportunity to better understand the process of CIOs’ role exit and CDOs’ role entry, and might inform about governance mechanisms and human resource management practices that contribute or impede a successful transition from an ambidextrous CIO role to the demand-side oriented role of CDOs and the supply-side oriented role of agile IT directors.

Aside from CDOs and CIOs, future research on the CEO-TMT interface can include chief innovation officers, CMOs, or chief strategy officers as these executives seem increasingly involved with digital technologies (Brinker and McLellan 2014; Taylor and Vithayathil 2018; Singh et al. 2020). Further studies on functional, structural, or social processes between CEOs, CDOs, and other TMT members might, for example, reveal information about governance for effective collaboration between CEOs and all TMTs involved with digital strategy development. Besides looking at shared understanding between CxOs (Onay et al. 2018), a “role-taking” lens (Georgakakis et al. 2019) might inform about how relational processes, e.g., trust between CEO and, e.g., CDO-CMO dyads impacts digital innovation performance. Further observing interactions between CEOs, CDOs, and other TMT members from a “role-multiplicity” perspective (Sieber 1974; Georgakakis et al. 2019) can reveal how digital strategists’ multiple roles might shape the interaction between CEOs, CDOs, and other TMT members.

### Consequences of CDO presence

Regarding the consequences of CDO presence, future research may further examine the all-important links between CDO presence and potential intermediate and final outcomes. Specifically, we encourage scholars to test whether links between CDO presence, intermediate, and final outcomes are in fact causal, i.e., whether appointing a CDO is conducive to successful digital transformation (and under which conditions) (Antonakis et al. 2010). In a similar vein, future research may also further explore the signaling function of CDO announcements. Scholars propose that companies hire CDOs to signal digital transformation effort to investors, but CDO announcements are not always positively associated with rising stock prices (Drechsler et al. 2019). Qualitative studies could explore potential reasons for stock price movements after CDO announcements. Interviewing investors might reveal whether CDO announcements in fact lead to changing stock prices due to more favorable investor perceptions or if omitted variables not considered in event studies may have caused price movements.

It may also be promising to take a glance at further outcomes of CDOs’ presence, e.g., CDOs’ impact on strategic actions like alliances with other firms, mergers and acquisitions, or changes to an organization’s structure (Nadkarni and Narayanan 2007). As extant research proposes a potential link between CDO presence in banks and increasing alliance behavior with fintechs (Hornuf et al. 2020), future studies may also seek to validate whether this link is causal and whether this behavior generalizes to contexts outside the banking sector.

Finally, there are several unanswered questions relating to the feedback effects of CDOs’ achievements on CDOs’ role. Cases of CDOs’ succession into CEO positions (Bain Capital and the CDO Club 2018) and consulting firms’ reports on CDOs’ possible temporariness suggest that the CDO position might disappear in some or even all firms over time. As the debate on CDOs’ potential disappearance is currently largely based on practitioner opinions and consulting reports, there are many opportunities for scholars to explore whether CDOs remain or disappear. In examining the antecedents and consequences of CDOs’ disappearance, researchers can further illuminate problems that might arise if nobody fills the role of a digital strategist, or opportunities that appear for other executives who might take over CDOs’ tasks.

### Contingency factors

Other fields of interest concern contingencies that might moderate various effects impacting or being caused by CDOs, for example, CDO hiring, CDOs’ scope of action, and CDOs’ impact on digital innovation and firm performance. Thus far, scholars have observed that the frequency of CDO recruitment seems to differ between different types of industries, e.g., between retail and financial sectors (Firk et al. 2019). Yet, it seems unclear why CDO recruitment has slowed down in certain industries (e.g., retail) but accelerated in others (e.g., financial). Future studies can compare CDO presence and effectiveness between different types of industries, e.g., between manufacturing and service industries, between high and low capital-intensive industries, or between high versus low technology-oriented sectors. Such studies can reveal further factors that may determine whether CDOs are a viable strategic choice only in specific industries.

More specifically, scholars can observe the fit between CDOs’ strategic decisions and industry clockspeed, i.e., the rate of industry change in terms of product innovations, process replacements, strategic shifts (e.g., alliances, mergers, acquisitions), or structural change (e.g., changes in top management). In line with Nadkarni and Narayanan (2007), who propose that the fit between industry clockspeed and top managers’ cognition affects strategic performance, observations on industry clockspeed may on the one hand inform whether CDOs meet the cognitive requirements for digital transformation in the respective industry. On the other hand, such research might reveal whether other TMT members, e.g., CIOs or CMOs, are more suitable for the job. In addition, analyzing samples of public, non-profit, and healthcare institutions might show if findings from large, publicly traded companies are generalizable to contexts in municipal governments, universities, or hospitals, which also hire CDOs (Krigsman 2015; CDO Club 2018, 2020b).

As CDOs are prevalent across geographies, observing country-specific factors, e.g., culture, economic development, or regulatory frameworks (Menz 2012) may generate insights into how globally operating firms can integrate CDOs in diverse geographic contexts. So far, Firk et al. (2019) find that country-specific institutional setups in their sample of Western publicly traded companies seem to affect CDOs’ presence. Moreover, the authors state that CDO presence varies between countries. Extending qualitative research on country-specific contingencies of CDO presence can shed light on how the geographical context determines CDOs’ endeavors and effectiveness. Additionally, extant research has mainly collected data in Europe or North America. As CDOs also appear in Russian and Asian contexts (Chhachhi et al. 2016; Rozanova 2019), considering data from Eastern nations and analyzing such data from a cultural perspective (e.g., House 2011) can serve as a starting point to account for country-specific factors that may be absent in Western countries.

It may also be promising to further assess how the organizational context affects CDOs’ role and effectiveness. Quantitative studies might assess the generalizability of qualitative findings on company size, a contingency factor that was proposed to determine CDOs’ scope of action (Horlacher et al. 2016; Becker et al. 2018). Moreover, qualitative studies on degrees of diversification or corporate culture, might reveal additional factors that affect the CDO role.

In addition, identifying contextual differences between companies that employ CDOs and show different levels of digital innovation performance might further inform about organizational contexts facilitating CDOs’ effectiveness. For example, a QCA approach could be useful in examining organizations with CDOs and trying to uncover whether, e.g., small company size, direct CDO-CIO reporting, or a centralized CDO position represent shared causal conditions between cases of high digital innovation performance. In light of the controversial discussions about the CDO role, such studies might highlight options for IS governance that increases CDOs’ effectiveness, and might quiet voices that claim that CDOs are “doomed to fail,” by showing that the failing cases were set up to fail from the beginning due to contextual factors (Walde 2020).

Contingencies that relate to the board of directors and its impact on strategic decision-making might further affect CDOs’ effectiveness. Aside from observing how the age of directors affects the likelihood of CDO hires (Kunisch et al. 2020), extant CDO research has largely ignored how board structure, composition, or processes influence CDOs’ activities and strategic decisions. As prior studies on board involvement in strategic decision-processes propose relationships between, e.g., board size or friendship ties between board members and the board’s involvement in strategic decisions (Finkelstein et al. 2009), we encourage future researchers to include board composition and activities as moderators of CDOs’ activities and their impact on digitization-related firm-level outcomes.

## Limitations and conclusions

As every literature review does, ours has several limitations. First, the results are based on keywords that represent existing labels for CDOs in studies published until July 2020. Although we followed the process suggested in the literature (e.g., Webster and Watson 2002; Tranfield et al. 2003; Short 2009) and we included journal articles across disciplines as well as conference proceedings, the results are limited by the scope of chosen keywords and the time-span of our search. Second, we only included English-language articles although CDOs are a global phenomenon and literature may exist in other languages and might, for instance, account for cultural aspects beyond the Western world. Aside from these limitations, we hope that our article encourages scholars to extend research on CDOs and thus contributes to our understanding of emerging top executives that guide digital strategy.

The article’s objective was to offer a review and research agenda on the emerging role of the CDO, a top executive role whose contribution to digital transformation is hotly debated among practitioners and researchers alike. Our study synthesizes the disconnected literature on CDOs and reveals promising insights into antecedents of CDO presence, CDO’s role, and CDOs’ impact on business digitization and firm performance. Building on our literature analysis, we propose a research agenda to not only answer remaining questions regarding CDO’s presence, role, and impact, but to also observe contingencies that might reveal information about additional factors that contribute to CDOs’ success, their potential disappearance, and consequences of their succession to CEO roles.

For managerial practice, our review summarizes the current scientific knowledge on CDO appointments, governance, and performance. Specifically, findings on antecedents of CDO presence as well as on CDOs’ professional backgrounds and skills can inform recruiting practices and help human resource professionals identify fitting candidates for the management of digital strategy. Regarding governance, practitioners can consider our findings on CDOs’ hierarchical position, horizontal governance, and reporting structures to improve the collaboration between existing CDOs, other TMT members, and employees. CEOs looking for performance measures to evaluate their CDOs’ effectiveness can implement and extend the suggested KPIs. Ultimately, our review of CDOs’ prototypical roles offers an overview of action fields where CDOs can innovate processes, coordinate digital transformation across departments, and overall, contribute to digital innovation performance.

## Supplementary Information

Below is the link to the electronic supplementary material.

Supplementary file1 (PDF 139 kb)

## Footnotes

1 We did not search for the term “CDO,” because CDO also abbreviates other, irrelevant phrases, e.g., “Collateralized Debt Obligations.” We thus assume that authors of works on Chief Digital Officers are likely to refrain from using *only* the abbreviation CDO in their studies.

2 We repeated the search in WoS, AISeL, IEEE Xplore, and EBSCO with the keywords “digital* transformation* officer*,” “chief digital* evangelist*,” “digital* director*,” “digital* officer*,” “group* digital* director,” “head* of digital*,” head* of digitalization*,” “SVP* digital*,” “vice president* digital*,” “head* digital*,” “global* head* of digital*,” “chief digital* marketing* officer*,” “digital* platform* officer*,” “ED-digital*” (suggested by Gimpel et al. 2018; Drechsler et al. 2019; Firk et al. 2019; Kunisch et al. 2020).
