# Supplementary material for: C﻿hief digital officers: the state of the art and the road ahead
Source: Manag Rev Q. 2021 Jun 7;72(4):1249–86. doi: 10.1007/s11301-021-00227-8 (PMC8182361; doi:10.1007/s11301-021-00227-8)
Supplement: Supplementary file 1 — Supplementary file1 (PDF 139 kb) [file 11301_2021_227_MOESM1_ESM.pdf]

Appendix

Table A1 Overview of Included Articles

Study	Database	Outlet	Major Theoretical Lens(es)	Research Design	Major Findings/Propositions	Theme(s) in Organizing Framework
**Becker et al. 2018**	AISeL	Proceedings of the Hawaii International Conference on System Sciences	Role Theory	Qualitative: Interviews with CDOs and other TMT members in 16 small, medium-sized, and large companies in multiple industries	Proposes that CDOs report all activities to the board.<br>Finds that CDOs' role varies within companies of different size.	CDO, Other TMT Members, Staff: Collaboration; Organizational Context
**Berman et al. 2020**	EBSCO	Strategy & Leadership	No information	Mixed Method: Survey with 750 CDOs and 750 other business executives in 23 countries and 18 industries, and interviews with CDOs	Finds that CDOs frequently have a business and/or technology background, and visionary skills.<br>Finds that CDOs frequently report to CEOs.<br>Proposes that reporting structures, CDOs' business background, and CDOs' strategic choices affect firm performance.	CDO Types: Professional Background and Skills; CDO, Other TMT Members, Staff: Collaboration; Final Outcomes: Financial Performance
**Buchwald and Lorenz 2020**	AOM Proceedings	Academy of Management Proceedings	Organizational Ambidexterity, Shared Understanding, Theory of Transactive Memory Systems	Conceptual	Proposes that the CDO and CIO roles reflect organizational ambidexterity.<br>Suggests that CDOs and CIOs roles can complement each other while their positions differ in terms of reporting structures, task focus, and role expectations.	Antecedents of CDO Presence: Individual; CDO, Other TMT Members, Staff: Potential Role Overlaps, Collaboration
**Drechsler et al. 2018**	AISeL	Proceedings of the Pacific Asia Conference on Information Systems	Role Typologies for IS Executives	Quantitative: Longitudinal study of global, publicly listed large manufacturers (2000 – 2016, archival data)	Intends to explore how CDOs in the role of innovation champions make use of internal reorganization, external knowledge recombination, and previous experience, to enhance digital innovation success.	CDO Types: Prototypical Roles

Study	Database	Outlet	Major Theoretical Lens(es)	Research Design	Major Findings/Propositions	Theme(s) in Organizing Framework
**Drechsler et al. 2019**	AISeL	Proceedings of the International Conference on Information Systems	Signaling Theory	Quantitative: Event study with 101 CDO announcements in publicly listed companies in Europe and North America (2002 – 2018, archival data)	Finds that CDOs' business background positively affects market-value.<br>Proposes that investors value CDO appointments in companies without CIO.<br>Proposes that CDO hires can serve as a positive signal to investors, but irrational CDO appointments (e.g., as a result of mimicry) may hamper performance.	Final Outcomes: Financial Perspective
**Firk et al. 2019**	AOM Proceedings	Academy of Management Proceedings	Contingency Theory	Quantitative: Longitudinal study of 919 international S&P 500 and MSCI Europe firms (2010 – 2017, archival data)	Proposes that firm size and product market diversification can lead to CDO presence.<br>Proposes that firms who are dependent on intangibles seem more likely to appoint CDOs than firms dependent on tangibles.<br>Finds that an increasing number of digital competitors increases CDO presence.<br>Proposes that intra-industry mimicry drives CDO presence.<br>Finds that country-specific institutional settings impact CDO presence.<br>Finds that CDOs' diffusion varies between countries.<br>Finds that CDO presence in companies dependent on intangibles, and high degrees of internal diversification increases financial performance.<br>Finds that low competition and low levels of country-specific digital readiness weakens the positive effect of CDO presence on financial performance.	Antecedents of CDO Presence: Firm, Environment; Final Outcomes: Financial Perspective
**Gerth and Peppard 2016**	WoS	Business Horizons	No information	Mixed Method: Interviews with 130 CDOs, CIOs, or non-IT executives, and survey with 675 CDOs in global firms	Finds that CDOs are appointed if executives perceive their CIOs as unable to foster digital strategy development.<br>Finds that companies appoint CDOs to react to digital competitors.<br>States that CDOs are unnecessary if CIOs are adequately set up for the job of digital strategists.	Antecedents of CDO Presence: Individual, Environment; CDO, Other TMT Members, Staff: Potential Role Overlaps

Study	Database	Outlet	Major Theoretical Lens(es)	Research Design	Major Findings/Propositions	Theme(s) in Organizing Framework
**Giebe 2019**	EBSCO	Journal of Economic Development, Environment and People	No information	Conceptual	Proposes that CDOs focus on promoting or coordinating digital innovation across functions.<br>Suggests that CDOs should focus on customers.<br>Finds that CDO presence in German banks is low compared to CDO presence in other industries.<br>Proposes that CDOs are not a good solution for German banks.	CDO Types: Prototypical Roles, Final Outcomes: Feedback-Effects on CDO Presence
**Gimpel et al. 2018**	AISeL	Journal of Information Technology Theory and Application	No theoretical lens	Qualitative: Exploratory interviews with CDOs, or other IS and business executives in 21 globally operating organizations of different size in multiple industries	Proposes that CDOs need ambition to promote a digital mindset.<br>Proposes that the choice for a CDO depends on the availability of other digital leaders.<br>Proposes that CDOs may become redundant once companies are digitized.	CDO Types: Professional Background and Skills; CDO, Other TMT Members, Staff: Potential Role Overlaps; Final Outcomes: Feedback-Effects on CDO Presence
**Haffke et al. 2016**	AISeL	Proceedings of the International Conference on Information Systems	Organizational Ambidexterity	Mixed Method: Exploratory interviews with CDOs or CIOs in 19 large or very large European firms in multiple industries, and survey with business and IT executives in the firms of the interview study	Finds that factors that concern individuals, the entire organizations, or the environment, alone or in combination, can lead to CDO presence.<br>Finds that CIOs' reputation matters when CDOs are appointed.<br>Finds that internal organizational complexity, digitization focus area, and competitors' pressure can lead to CDO appointments.<br>Finds that CDOs interact within "digital innovator," "digital evangelist," "digitization coordinator," and "digital advocate" role profiles.<br>Finds that CDOs represent a transition of the CIO role.<br>Proposes that some CDOs might disappear or get promoted to other executive roles.	Antecedents of CDO Presence: Individual, Firm, Environment; CDO Types: Prototypical Roles; CDO, Other TMT Members, Staff: Potential Role Overlaps; Final Outcomes: Feedback-Effects on CDO Presence

Study	Database	Outlet	Major Theoretical Lens(es)	Research Design	Major Findings/Propositions	Theme(s) in Organizing Framework
**Hansen and Sia 2015**	Backward	MIS Quarterly Executive	No theoretical lens	Qualitative: Exploratory interviews with CEO, CMO, chief sales officer at a European sports fashion retailer	Proposes that CIOs with business and marketing expertise can step into the CDO role.<br>Proposes that CDOs' role might disappear.	CDO, Other TMT Members, Staff: Potential Role Overlaps; Final Outcomes: Feedback-Effects on CDO Presence
**Horlacher and Hess 2016**	IEEE	Proceedings of the Hawaii International Conference on System Sciences	Concepts for Managerial Roles; Organizational Ambidexterity	Qualitative: Interviews with four CDOs in four companies of different size in multiple industries	Finds that CDOs can interact within the roles of an "entrepreneur", "spokesperson," "leader," or "liaison."<br>Finds that CDOs and CIOs are complementary actors.<br>Finds that CDOs' role varies between large and small companies.	CDO Types: Prototypical Roles; CDO, Other TMT Members, Staff: Collaboration; Organizational Context
**Horlacher 2016**	AISeL	Proceedings of the European Conference on Information Systems	Concepts of Shared Understanding; Theory of Transactive Memory Systems	Mixed Method: Interviews with CDOs and CIOs in four companies of different size in different industries	Finds that clear role divisions, regular meetings, and similar professional and educational background between CDOs and CIOs foster their successful collaboration.	CDO, Other TMT Members, Staff: Collaboration
**Horlacher et al. 2016**	AISeL	Proceedings of the Americas Conference on Information Systems	Vertical and Horizontal IT Governance Mechanisms	Qualitative: Interviews with CDOs, senior IT executives, managing director in three medium or large companies in different industries	Finds that the interplay of vertical and horizontal governance mechanisms affects CDOs' scope of action.	CDO Types: Hierarchical Position; CDO, Other TMT Members, Staff: Collaboration

Study	Database	Outlet	Major Theoretical Lens(es)	Research Design	Major Findings/Propositions	Theme(s) in Organizing Framework
**Hornuf et al. 2020**	WoS	Small Business Economics	No Specific Theoretical Lens	Quantitative: Longitudinal study of the 100 largest banks in Canada, France, Germany, United Kingdom (2007 – 2017, archival data)	Proposes that CDO presence/and or a focus on digital strategy might be positively related to banks' alliances with fintechs.	Intermediate Outcomes: Digitalization-related
**Kunisch et al. 2020**	Expert	Long Range Planning	Contingency Theory	Quantitative: Longitudinal study of 1,755 US S&P 1500 companies (2000 – 2018, archival data)	Finds that CDOs' diffusion accelerated between 2010 and 2018 in S&P 1500 firms.<br>Proposes that a lack of digital competence as well as board composition affects CDO presence.<br>Finds that firm size, firm focus, and sales ratios affect CDO presence.<br>Proposes that mimicry drives CDO presence.<br>Finds that CDOs can have specialist and generalist roles.	Antecedents of CDO Presence: Individual, Firm, Environment; CDO Types: Prototypical Roles
**Leonhardt et al. 2018**	AISeL	Proceedings of the International Conference on Information Systems	Configurational Theory; Vertical and Horizontal IT Governance Mechanisms	fsQCA: Survey of 222 IT managers in organizations of different size and industries in the US	Finds that CDOs provide opportunities for digitalization-related performance if other organizational bodies are not in charge of digital projects.	Intermediate Outcomes: Digitalization-related
**Moker 2020**	AISeL	Proceedings of the European Conference of Information Systems	Upper Echelon Theory	Quantitative: Longitudinal study of largest firms on the German stock index HDAX (2011 – 2019, archival data)	Intends to explore the impact of CDOs' professional background and career experience on digital transformation initiatives.	CDO Types: Professional Background and Skills

Study	Database	Outlet	Major Theoretical Lens(es)	Research Design	Major Findings/Propositions	Theme(s) in Organizing Framework
**Onay et al. 2018**	AISeL	Proceedings of the International Conference on Information Systems	Theory of Shared Understanding	Mixed Method: Survey of CEOs, CIOs, CDOs and other CxOs	Intends to observe the effect of shared understanding between several CxOs on IT alignment.	CDO, Other TMT Members, Staff: Collaboration
**Reck and Fliaster 2018**	AOM Proceedings	Academy of Management Proceedings	Contingency Theory; Configurational Theory	fsQCA: Survey of 96 CDOs in 1500 manufacturing firms of several sizes in Germany, Austria, and Switzerland	Finds that CDOs interact within the roles of a “process promoter,” “relationship promoter,” or “innovation champion.”<br>Proposes several solutions for configurations of CDO type, CDOs’ influence, and competitive pressure, that lead to digital innovation performance.	CDO Types: Prototypical Roles; Intermediate Outcomes: Digitalization-related
**Reck and Fliaster 2019**	EBSCO	MIT Sloan Management Review	Contingency Theory; Configurational Theory	fsQCA: Survey of 97 digital leaders in manufacturing companies of various sizes in Germany, Austria, and Switzerland	Finds that CDOs interact within the roles of an “insider expert,” “networker and catalyzer,” “innovation evangelist,” or “lone icebreaker.”<br>Proposes several solutions for configurations of CDO type, CDOs’ influence, and competitive pressure, that lead to digital innovation performance.	CDO Types: Prototypical Roles; Intermediate Outcomes: Digitalization-related
**Seeher et al. 2020**	AISeL	Proceedings of the European Conference of Information Systems	No information	Delphi Study: Survey with 38 current or former CDOs, consultants, academics, or other managers working with CDOs	Proposes KPIs for the CDO types “marketer,” “orchestrator,” “evangelist,” and “innovator.”	CDO Types: Prototypical Roles

Study	Database	Outlet	Major Theoretical Lens(es)	Research Design	Major Findings/Propositions	Theme(s) in Organizing Framework
**Singh and Hess 2017**	WoS	MIS Quarterly Executive	No information	Qualitative: Interviews with CDOs in six companies of different size and industry	Finds that great internal complexity and high market pressures drive CDO presence.<br>Finds that CDOs interact within the primary roles of an "evangelist," "coordinator," or "entrepreneur."<br>Finds that CDOs need skills in business and IT, and various soft skills.<br>Finds that CDOs and CIOs have distinct task foci.<br>Proposes that CDOs might disappear or get promoted to further executive roles.	Antecedents of CDO presence: Firm, Environment; CDO Types: Prototypical Roles, Professional Background and Skills; CDO, Other TMT Members, Staff: Potential Role Overlaps; Final Outcomes: Feedback-Effects on CDO Presence
**Singh et al. 2020**	WoS	Long Range Planning	Vertical and Horizontal IT Governance Mechanisms	Qualitative: Interviews with CDOs, and other IS executives and business executives in four companies of different size in four different industries operating globally	Highlights the importance of aligning vertical and horizontal governance mechanisms with the scope of digital transformation strategy, and CDOs' task focus.	CDO Types: Hierarchical Position; CDO, Other TMT Members, Staff: Collaboration
**Tahvanainen and Luoma 2018**	AISeL	Proceedings of the Americas Conference on Information Systems	Competence Framework for IT Professionals	Qualitative: Exploratory interviews with ten CDOs in companies of different size in multiple industries in Nordic countries	Finds that CDOs need multidisciplinary professional skills in business and IT, as well as various soft skills.	CDO Types: Professional Background and Skills

Study	Database	Outlet	Major Theoretical Lens(es)	Research Design	Major Findings/Propositions	Theme(s) in Organizing Framework
**Tumbas et al. 2017**	WoS	MIS Quarterly Executive	No theoretical lens	Qualitative: Exploratory interviews with 35 CDOs from several industries in North America, Europe, Australia, and South America	Finds that reasons to hire CDOs may be siloed or busy IT and marketing departments, or the lack of a company-wide digital strategy.<br>Finds that CDOs interact within the roles of a "digital marketer," "digital harmonizer," or "digital accelerator."<br>Proposes that the job title of CDOs might evolve into further roles, e.g., chief innovation officers.	Antecedents of CDO Presence: Firm; CDO Types: Prototypical Roles; Final Outcomes: Feedback-Effects on CDO Presence
**Tumbas et al. 2018**	WoS	Journal of Information Technology	Institutional Entrepreneurship, Logics of Action	Qualitative: Exploratory interviews with 35 CDOs from several industries and one interview with the founder of the CDO Club (a community for CDOs)	Finds that CDOs follow a digital logic of action while incumbent IT executives follow an IT logic of action.<br>Proposes that CDOs can use "grafting," "bridging," and "decoupling" strategies to navigate tensions when they enter incumbent IT departments with differing action logics.	CDO, Other TMT Members, Staff: Collaboration, Conflict
**Wade and Obwegeser 2019**	EBSCO	MIT Sloan Management Review	No information	Mixed Method: Survey of more than 1,000 CDOs and other digital leaders, and interviews with 42 digital leaders in several industries across 28 countries	Finds that CDOs' effectiveness depends on competences and credibility.<br>Distinguishes between CDO types that face low role ambiguity and CDO types that face high role ambiguity.<br>Proposes that functional CDOs can be hired externally, and cross-functional CDOs should be hired internally	CDO Types: Prototypical Roles; Professional Background and Skills

Study	Database	Outlet	Major Theoretical Lens(es)	Research Design	Major Findings/Propositions	Theme(s) in Organizing Framework
**Zhan and Mu 2019**	IEEE Xplore	Proceedings of the International Conference on Service Systems and Service Management	Role Ambiguity	Quantitative: Event Study with 97 CDO announcements in publicly traded firms in the US (2004 –2015, archival data)	Proposes that job overlaps between CDO and CIO lead to depressed stock prices.	CDO, Other TMT Members, Staff: Conflict; Final Outcomes: Financial Performance
